# Optimization of the fermentation media, mathematical modeling, and enhancement of paclitaxel production by *Alternaria alternata* after elicitation with pectin

**DOI:** 10.1038/s41598-024-63681-w

**Published:** 2024-06-05

**Authors:** Hamzeh Rezazadeh, Faezeh Ghanati, Mercedes Bonfill, Fatemeh Nasibi, Mehdi Tabarsa

**Affiliations:** 1 Department of Plant Biology, Faculty of Biological Sciences, Tarbiat Modares University (TMU), POB 14115-154, Tehran, Iran; 2 Department of Biology, Healthcare and the Environment, Faculty of Pharmacy and Food Sciences, University of Barcelona, Barcelona, Spain; 3 Department of Biology, Faculty of Sciences, Shahid Bahonar University of Kerman, Kerman, Iran; 4 Department of Seafood Processing, Faculty of Marine Sciences, Tarbiat Modares University, Noor, Iran

**Keywords:** *Alternaria alternata*, *Corylus avellana*, Endophytic fungi, Paclitaxel, Taxanes, Microbiology, Plant sciences

## Abstract

*Alternaria alternata* fungus is a potent paclitaxel producer isolated from *Corylus avellana*. The major challenge is the lack of optimized media for endophytic fungi productivity. In the effort to maximize the production of taxoids by *A. alternata*, several fermentation conditions, including pH (pH 4.0–7.0), different types and concentrations of carbon (fructose, glucose, sucrose, mannitol, sorbitol, and malt extract), and nitrogen (urea, ammonium nitrate, potassium nitrate, ammonium phosphate, and ammonium sulfate) were applied step by step. Based on the results, *A. alternata* in a medium containing sucrose 5% (w/v) and ammonium phosphate 2.5 mM at pH 6.0 showed a rapid and sustainable growth rate, the highest paclitaxel yield (94.8 µg gFW^−1^ vs 2.8 µg gFW^−1^ in controls), and the maximum content of amino acids. Additionally, the effect of pectin was evaluated on fungus, and mycelia harvested. Pectin significantly enhanced the growth and taxoid yield on day 21 (respectively 171% and 116% of their corresponding on day 7). The results were checked out by mathematical modeling as well. Accordingly, these findings suggest a low-cost, eco-friendly, and easy-to-produce approach with excellent biotechnological potential for the industrial manufacture of taxoids.

## Introduction

Taxanes are a diverse group of phytochemical compounds originally derived from *Taxus* species. Although their applications extend beyond cancer treatment, their primary focus is on inhibiting cell mitosis and stimulating apoptosis in various cancer cells. Evidence show that taxanes present a diverse array of compounds with distinct anticancer properties, offering promising avenues for targeted therapeutic interventions. Several key taxoids play significant roles in therapeutic endeavors. For instance, paclitaxel, the most well-known taxane has been used to treat cervical and ovarian cancer since 1986^1^. Antiviral activity of 10-deacetyl baccatin III and its inhibitory function on replication of Herpes simplex type 1 virus (HSV-1) have been recently shown^2^. Baccatin III, another taxoid worthy of note, has demonstrated therapeutic potential against idiopathic pulmonary fibrosis, a progressive and usually fatal lung disease^3^. Cephalomannine particularly targets lung cancer^4^. Research suggests that 7-epi-10-deacetyl paclitaxel has been identified for its ability to induce apoptosis in human hepatocellular carcinoma^5^.

The limited availability of natural sources of taxanes and environmental concerns about their scarcity motivated the search for alternative, sustainable, and scalable sources^6^. In this context, endophytic fungi, which reside within the tissues of various plant species, have emerged as promising candidates for producing paclitaxel and other taxanes^7^. These symbiotic microorganisms exhibit the intriguing ability to produce paclitaxel or its intermediates^8^, but it is unlikely to closely resemble the biosynthetic pathway found in *Taxus* species^9^.

In recent years, bioprospecting of fungal endophytes for new compounds has attracted greater attention^10,11^. Generally, endophytes proved to be excellent and sustainable sources of many bioactive compounds with potential applications in pest control^12^, bio-pigment production^13^, antibiotics^14^, antioxidant and antimycotic activities^15^, manufacturing terpenoids and other volatile organic compounds^16,17^, food industry, and other pharmaceutical industry^18^. *Strobel* et al., also demonstrated that certain endophytic fungi can introduce to alternative and sustainable sources of fuel-related hydrocarbons^19^.

*Shrestha *et al., have identified endophytic fungi as promising candidates for the pharmaceutical industry, particularly for the production of vital medications such as paclitaxel^20^, which offers a groundbreaking solution to address production chain limitations and propel advancements in its strategic manufacturing. Endophytic fungi offer several advantages, including rapid growth, ease of cultivation, and potential genetic manipulation, making them attractive candidates for paclitaxel production^21^. Extensive research has been devoted to isolating and characterizing a diverse array of endophytic fungi capable of paclitaxel biosynthesis^22^, with the ultimate goal of developing sustainable and scalable production methods^23^.

In the quest to enhance paclitaxel production, various approaches have been explored. These encompass the application of environmental stimuli e.g. temperature, drought, light, and pressure, and feeding with minerals, sugars, amino acids, and vitamins^24,25^.

Each of these approaches holds great promise but also presents a set of inherent limitations. For instance, the availability and cost of specific hormones for biological signaling may vary. Excessive temperatures and drought may negatively impact enzymes and the growth and survival of microorganisms^26^. High-intensity light stress can result in photo-inhibition and cellular damage^27^, and pressure stress may require specialized equipment. Genetic manipulation can sometimes result in unintended consequences such as alteration of natural traits and also may pose ethical hurdles^28^. Suboptimal nutrient composition potentially leads to uneven growth and reduces secondary metabolite production. Optimization of medium nutrients, however, represents a potent and versatile strategy for enhancing the growth of microorganisms. Meticulous adjustment of culture composition through different carbons and nitrogen sources and pH optimizes the growth of microorganisms and the production of secondary metabolites simultaneously^29,30^.

**Figure 1:**
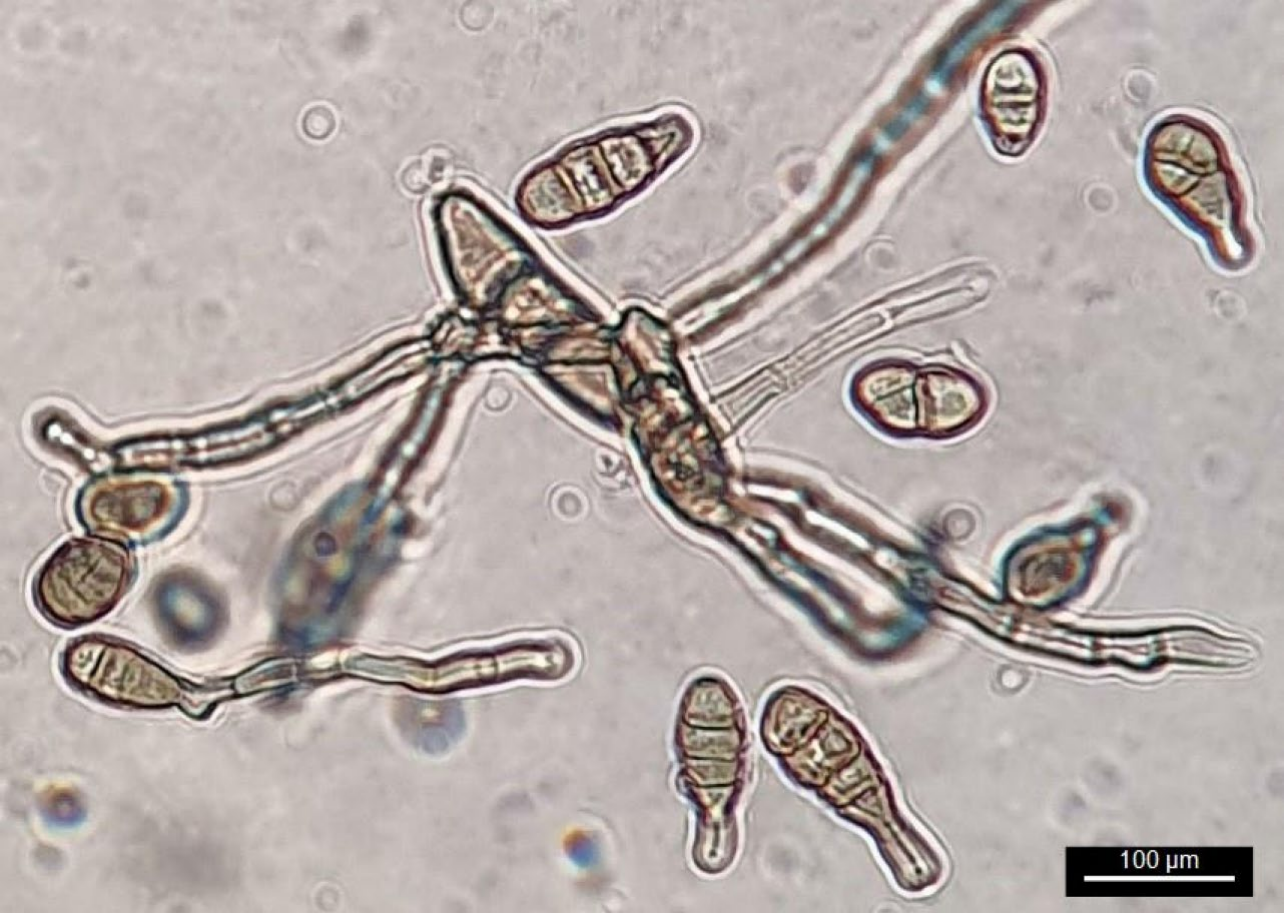
Morphology of the multicellular conidia of *A. alternata*. The fungus was grown on PDA and incubated at 25 °C in dark.

There is a hypothesis that endophyte-host interactions involve constant mutual antagonism at least in part based on the secondary metabolites the partner produces^31^. Such mutualistic relationships are mediated by certain molecules known as elicitors. A growing body of evidence has verified the positive effects of pectic poly- or oligosaccharides in elicitation defense response and improvement of secondary metabolite biosynthesis. The subject of the current research is the optimization of the culture composition to achieve sustainable growth accompanied by reproducible taxane production at a constant rate by *A. alternata* (Fig. 1) isolated from *Corylus avellana*. Also, to the best of our knowledge, few experiments have surveyed the elicitation effects of host polysaccharides on taxane production by endophytes. The feasibility of application of host pectin as a stimulant of growth and taxane yield by the fungus was also investigated.

## Results

### Total taxoid yield and growth rate of *A. alternata* in the different media

**Figure 2:**
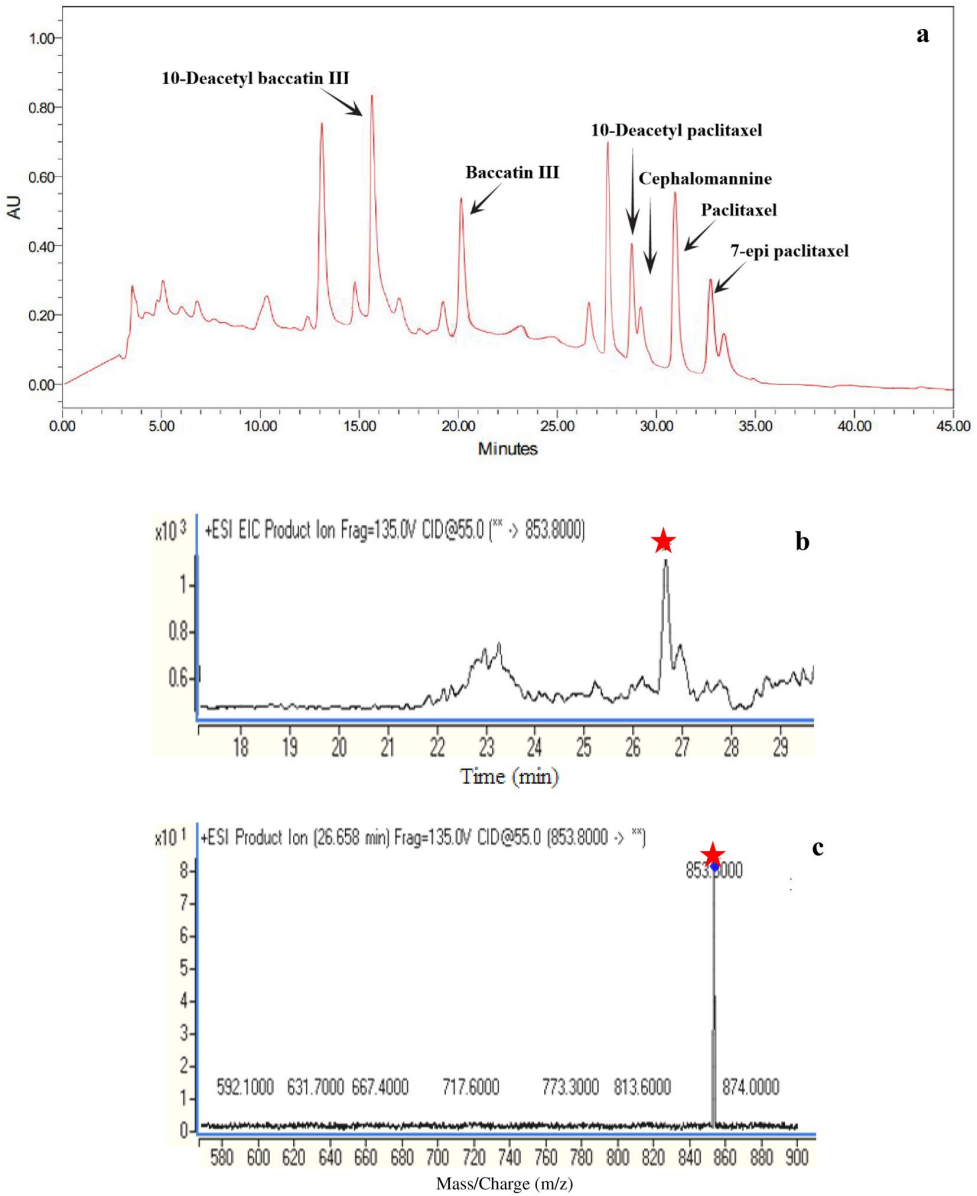
Chromatogram of *A. alternata* extract analyzed by HPLC and LC–MS. Peaks at 15.6, 20.2, 28.7, 29.3, 31.2, and 32.8 min are corresponding to 10 deacetyl baccatin III, baccatin III, 10-deacetyl paclitaxel, cephalomannine, paclitaxel, and 7 epi-paclitaxel, respectively (**a**). The asterisk denotes the presence of paclitaxel in the sample analyzed by LC–MS (**b**), with ion mass spectrum shown in sample (**c**).

The presence of paclitaxel and other taxanes was monitored via HPLC analysis of the extract of isolated fungus (Fig. 2a). The structure of the detected paclitaxel was confirmed by LC–MS. The [M+H] peak was considered at 853 m/z for paclitaxel in fungus extract (Fig. 2b), which was corresponding to the same peak in standard of paclitaxel (Fig. 2c).

**Figure 3:**
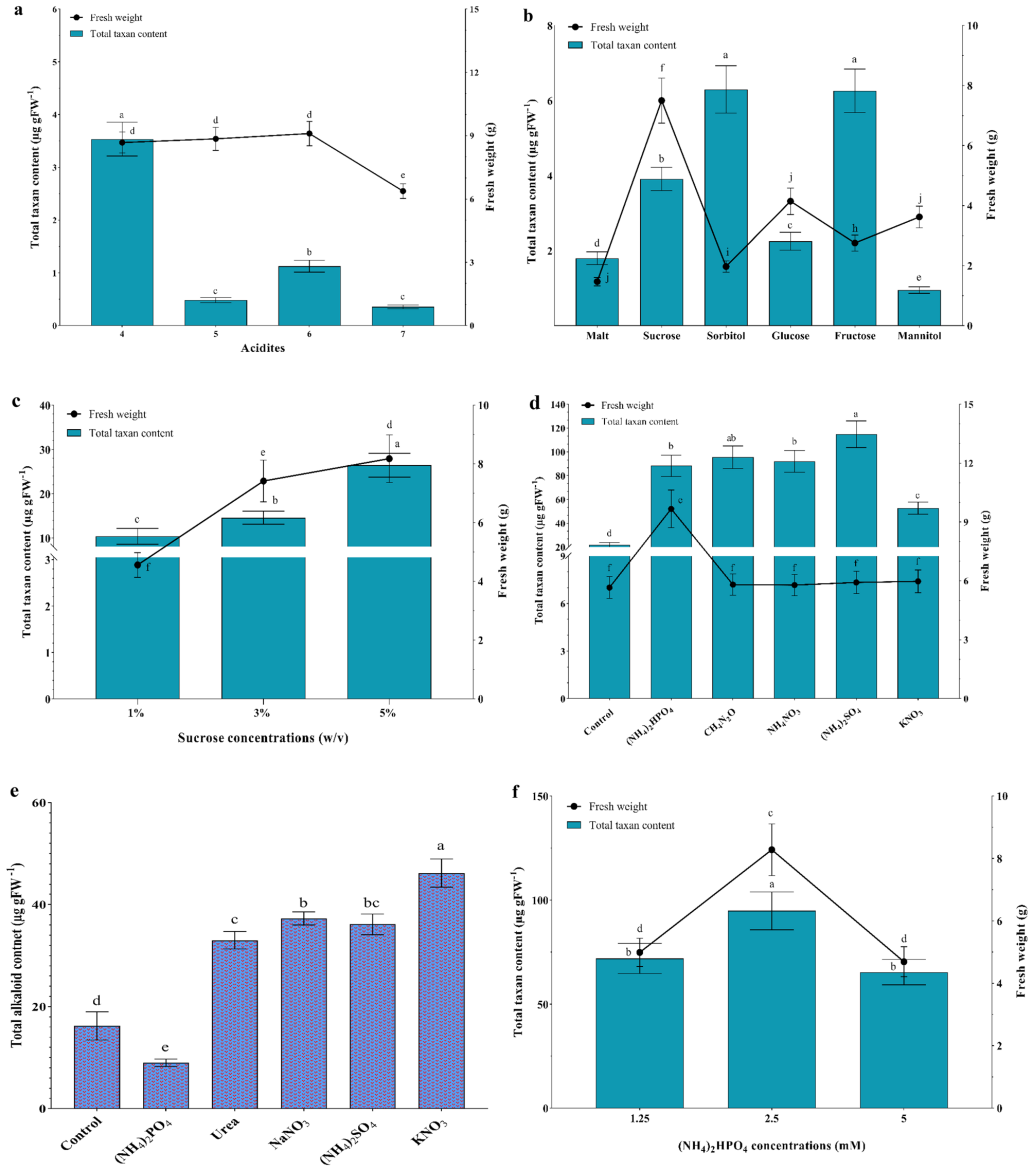
Effects of acidity, carbon, and nitrogen types on growth characteristics, and total taxane content of *A. alternata*. Acidity (**a**), carbon sources (**b**), various concentrations of sucrose (**c**), nitrogen sources (**d**), total alkaloid content (**e**), and different concentrations of ammonium phosphate (**f**). Data indicate mean ± SD, n = 3. Different letters show significant differences at *p* ≤ 0.05 based on Duncan’s analyses.

The influence of varying acidity levels, carbon source, and nitrogen source on the total taxane content, and growth rate of *A. alternata* are illustrated in Fig. 3. In the initial stages of fungus culture in potato dextrose broth (PDB) media, the fungus biomass at pH 4.0, 5.0, and 6.0 exhibited no significant differences, while a notable reduction in the biomass at pH 7.0 was observed (Fig. 3a). At pH 4.0, the highest total taxoid content was observed; however, pH 5.0 and pH 7.0 showed the lowest amount of taxane (Fig. 3a).

As shown in Fig. 3b, in pursuit of attaining the maximizing taxane yield and high growth rate, targeted research was conducted on *A. alternata*. As carbon sources, when sorbitol and fructose were utilized, a significant increase in the total taxane was observed, but sucrose especially boosted the biomass of *A. alternata* (Fig. 3b).

According to Fig. 3c, the comparative analysis of the taxoid content and growth rate underscores that sucrose 5% serves as a suitable concentration of favorable carbon source for *A. alternata* (Fig. 3c).

The application of exogenous nitrogen in the different forms substantially influenced the total taxane yield of *A. alternata* (Fig. 3d), and all nitrogen sources contributed to a noteworthy enhancement in total taxane content. The research showed ammonium phosphate as a nitrogen source can improve the growth rate of fungus, resulting in an approximate 70% increase compared to alternative nitrogen sources (Fig. 3d).

To further investigate the impact of nitrogen sources on fungus, their influence on the quantity of amino acids and total alkaloid contents was also assessed. The amount of amino acids in the fungus was outstandingly augmented when it was located in ammonium phosphate (up to almost 3.3 folds higher than the control) (Table 1). Histidine was the most abundant among the amino acids in *A. alternata* extract.

**Table 1:** Detected amino acids (µg gFW^−1^) of *A. alternata* in different nitrogen sources.

	Asparagine	Glutamine	Serine	Histidine	Arginine	Tyrosine	Methionine	Phenylalanine	Isoleucine	Leucine	Total aminoacids
	µg gFw^−1^										
Control	V. L	1.2 ± 0.25 d	V. L	13.2 ± 1.52 c	0.13 ± 0.002 c	0.22 ± 0.015 d	0.10 ± 0.05 d	0.11 ± 0.004 b	V. L	V. L	14.99 ± 1.84 c
KNO_3_	1.49 ± 0.06 a	6.64 ± 0.54 b	0.15 ± 0.003 b	0.38 ± 0.03 d	1.42 ± 0.02 a	1.98 ± 0.02 a	0.29 ± 0.03 b	0.15 ± 0.004 a	V. L	N. D	11.9 ± 0.7 d
NaNO_3_	0.44 ± 0.015 c	3.2 ± 0.16 c	V. L	21.43 ± 3.1 b	V. L	0.28 ± 0.02 c	V. L	V. L	V. L	N. D	25.67 ± 3.3 b
Urea	0.58 ± 0.026 b	0.85 ± 0.015 f.	V. L	23.64 ± 3.2 b	0.71 ± 0.01 b	0.41 ± 0.015 b	0.64 ± 0.04 a	V. L	V. L	N. D	26.87 ± 3.3 b
(NH_4_)_2_ HPO_4_	0.62 ± 0.02 b	10.58 ± 0.6 a	0.25 ± 0.004 a	53.51 ± 6.12 a	V. L	V. L	0.2 ± 0.02 c	0.13 ± 0.02 b	0.10 ± 0.04 a	N. D	65.40 ± 6.82 a
(NH_4_)_2_SO_3_	V. L	0.91 ± 0.01 e	V. L	12.42 ± 1.51 c	V. L	0.23 ± 0.015 d	0.16 ± 0.01 d	V. L	V. L	N. D	13. 86 ± 1.55 cd

Values are means of three replications ± standard deviation. Different letters show the significant difference with *p* ≤ 0.05 in one-way ANOVA and Duncan’s analyses. Means followed by the same letter in each column are not significantly different.

*V. L* very low, the content less than 0.1 µg gFW^−1^, *N. D* none detected.

The total content alkaloid in potassium nitrate source experienced a remarkable surge, reaching approximately 184% of the control. Noteworthy, when ammonium phosphate was used, the alkaloid content underwent a dramatic reduction (Fig. 3e).

Feeding *A. alternata* with ammonium phosphate 2.5 mM significantly affected the total taxane content. Of particular note is the most elevated biomass was achieved when using (NH_4_)_2_HPO_4_ 2.5 mM (Fig. 3f).

### The taxane profile in different media

**Figure 4:**
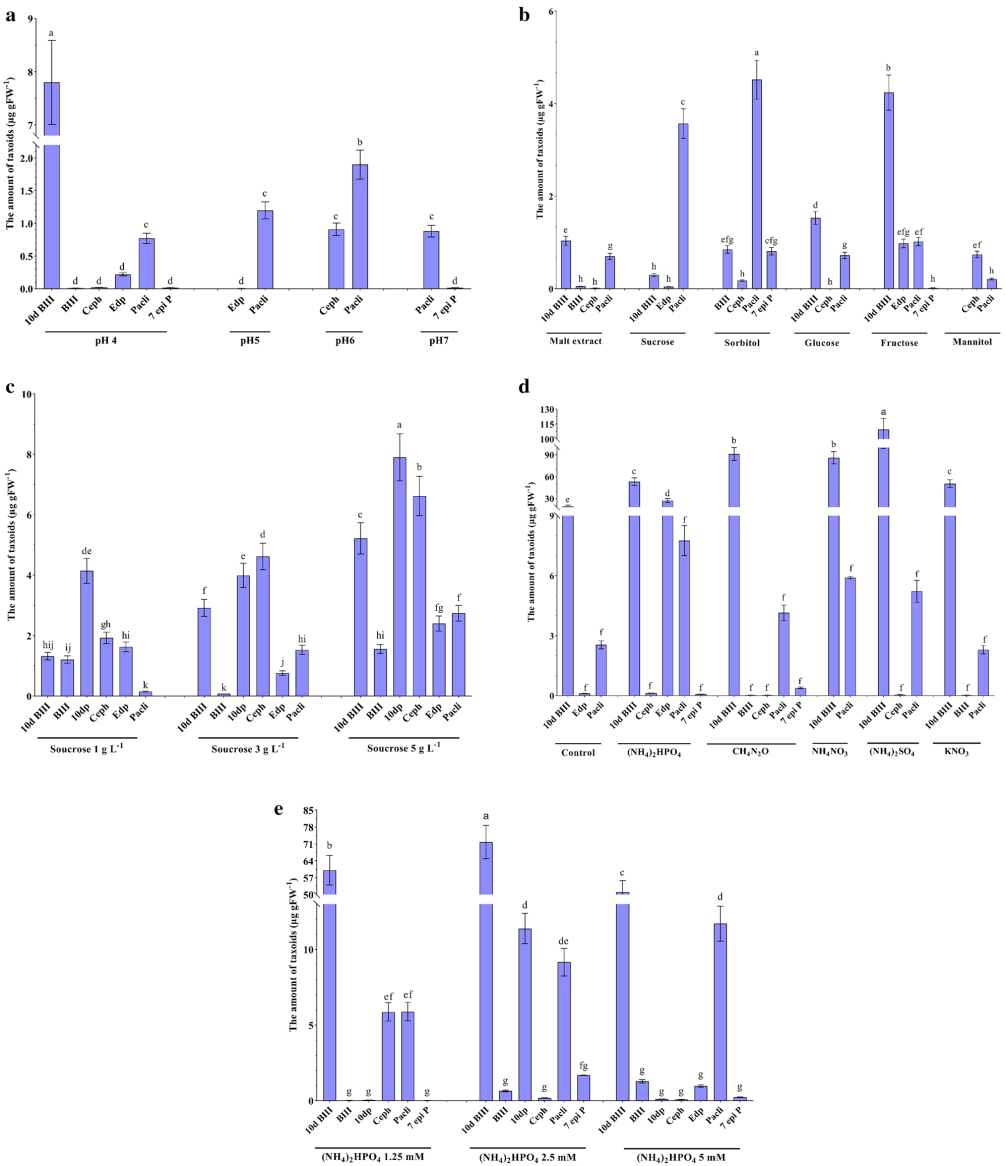
Taxane profiles of *A. alternata* by modifying the culture medium. Various acidities in PDB (**a**), carbon sources at pH 6.0 (**b**), various concentrations of sucrose (**c**), various nitrogen sources (**d**), the optimized medium containing 5% (w/v) sucrose in pH 6.0 (**e**). Data indicate mean ± SD, n = 3. Different letters show significant differences at *p* ≤ 0.05 based on Duncan’s analyses. 10-deacetyl paclitaxel (10dp), 10-deacetyl baccatin III (10d BIII), 7-epi paclitaxel (7 epi P), 7-epi-10-deacetyl paclitaxel (Edp), baccatin III (BIII), cephalomannine (Ceph), paclitaxel (Pacli).

Figure 4 illustrates the taxane profile synthesized by *A. alternata* under varying levels of acidities, carbon, and nitrogen sources. At the outset, Fig. 4a elucidates the effect of different acidity levels on taxanes. In PDB media with pH 4.0, six taxanes i.e., 10-deacetyl baccatin III, baccatin III, cephalomannine, 7-epi-10-deacetyl paclitaxel, paclitaxel, 7-epi paclitaxel were produced by the fungus. Notably, paclitaxel recognized as the principal end product in the taxane biosynthesis pathway, exhibited a significant increase at pH 6.0, reaching levels up to 1.5 times higher than this observed at pH 4.0. Following these observations and previous findings, further investigations were conducted to delve deeper into the factors influencing taxane production by *A. alternata* at pH 6.0 (Fig. 4a).

The data presented in Fig. 4b illustrates the taxane profile within a medium containing various carbon sources, followed by different concentrations of the carbon source (Fig. 4c). The least alterations in taxoids were noted when mannitol was utilized as the carbon source. Fructose notably enhanced the yield of 10-deacetyl baccatin III. When sucrose and sorbitol were utilized as carbon sources, there was a notable increase in the production of paclitaxel.

The yield of paclitaxel experienced a notable increase specifically at the 5% (w/v) sucrose concentration (Fig. 4c). Further investigations revealed the synthesis of six taxoid compounds in the medium with pH 6.0 and sucrose 5% (w/v), introducing three newly discovered ones: Baccatin III, 10-deacetyl paclitaxel, and cephalomannine (Fig. 4c). Consequently, diverse nitrogen source types were assessed in the 5% sucrose medium at pH 6.0 to expand the scope of the investigation.

The role of nitrogen as an exogenous force made transformative patterns in the taxane profile compared to the previous stage (Fig. 4d). In addition, nitrogen sources became evidence as enhancers of the taxane production. Under the influence of ammonium sulfate, 10-deacetyl baccatin III experiences a significant upswing, indicating a pronounced positive impact on the stages of taxane synthesis. Notably, ammonium phosphate takes center stage, orchestrating the amount increase of paclitaxel (up to 200%), compared to the control (Fig. 4d).

A closer examination of different concentrations of ammonium phosphate as a nitrogen source showed that not only the total amount of taxanes was decreased, but also the taxane profile underwent a remarkable change (Fig. 4e). Based on this investigation, the highest amount of paclitaxel was identified at the higher concentration (5 mM) of (NH_4_)_2_HPO_4_ (Fig. 4e).

### Efficacy of pectin on the growth rate, total content taxoids, terpenoids, and the profile of taxanes

**Figure 5:**
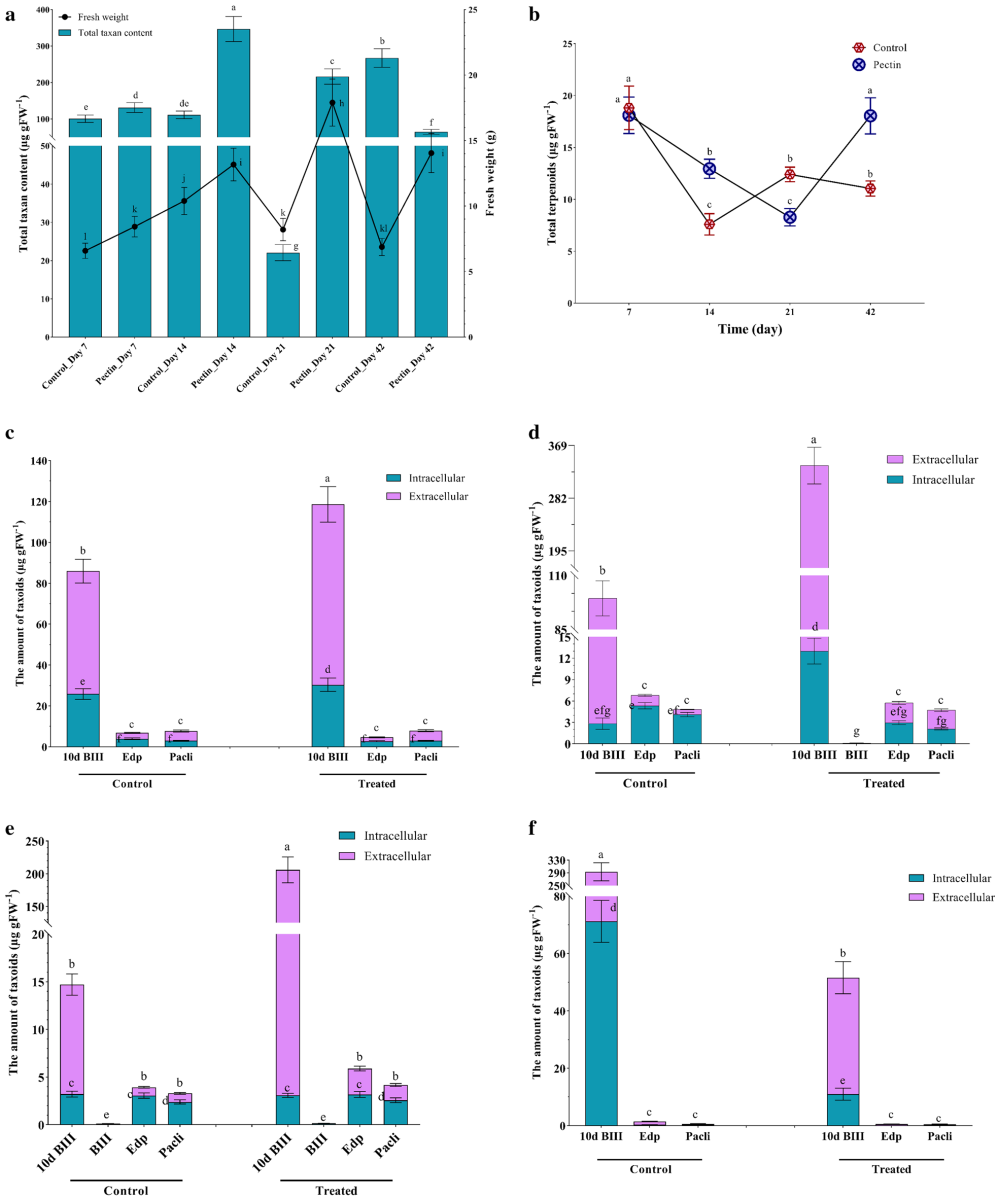
Effects of pectin on total taxane content and growth rate (**a**), terpenoid (**b**), and the profile of taxanes in the day 7 (**c**), 14 (**d**), 21 (**e**), and 42 (**f**). Data indicate mean ± SD, n = 3. Different letters show significant differences at *p* ≤ 0.05 based on Duncan’s analyses. 10-deacetyl baccatin III (10d BIII), 7-epi-10-deacetyl paclitaxel (Edp), baccatin III (BIII), paclitaxel (Pacli).

The optimized medium (i.e., pH 6.0, sucrose 5% (w/v), and ammonium phosphate 2.5 mM) was used to assess the effect of pectin on *A. alternata* for 42 days (Fig. 5). Using pectin as an elicitor introduces a dynamic shift in the growth pattern of the fungus across distinct time courses. As shown in Fig. 5a, the growth rate experienced a tangible surge up to the 21th day (approximately 118% of the control). However, the total taxane yield due to pectin stimulation on the 14th and 21th days was around 1.1 and 8 times higher than the control on the 14th and 21th days, respectively (Fig. 5a).

According to Fig. 5b, no significant difference in terpenoid yield was observed between the control and the treated samples on day 7 of the test (Fig. 5b). However, on the 21th day, the amount of terpenoids produced by the treatment decreased significantly compared to previous days. Conversely, a decline was observed on the 14th day for the control group (Fig. 5b).

According to Fig. 5c–f, several types of taxoid compounds were produced by *A. alternata* under the effect of pectin. The temporal evolution of these compounds reveals intriguing patterns. As a result of pectin's influence on the taxane profile, *A. alternata* possesses the ability to produce three key vineyard compounds: 10-deacetyl baccatin III, 7-epi-10-deacetyl paclitaxel, and paclitaxel on the 7th day (Fig. 5c). Also, a notable increase was observed in 10-deacetyl baccatin III on the 7th day, ca. 37% increase compared to the control (Fig. 5c).

As the timeline progresses to the 14th day, the compound 10-deacetyl baccatin III experiences a significant increase in the treated mycelia (up to almost 24 folds higher than the control) (Fig. 5d). Moreover, the treatment reveals a fascinating development—the emergence of one novel taxane, baccatin III on day 14. The maximum taxane production is observed on the 14th day, where 10-deacetyl baccatin III exhibits a substantial increase (approximately 250% higher than the control) (Fig. 5d). However, the profile of taxoids on days 21 and 42 indicates that they have been reduced under the pectin treatment (Fig. 5e,f).

The research illuminates an interplay between phenolic acids and the pectin elicitor according to Table 2. On the seventh day post-pectin induction, no change is seen in the content of phenolic acids. Interestingly, in the control group, the content of phenolic acids reaches its peak on the 42th day (up to 1.1 fold of the treatment on day 42). In contrast, for the pectin-affected cells, this scenario extended until the 21th day (approximately 25% of the control on day 21). (approximately 25% of the control on day 21). According to these results, the highest amount of 4-hydroxybenzoic acid was recorded on the 7th day of the control, while the highest amount of Rosmarinic acid was accumulated on the 42th day of the control. Additionally, Cinnamic acid was accumulated in *A. alternata* at the highest levels on days 42 and 21 of the control (Table 2).

**Table 2:** Detected phenolic compound (µg gFW^−1^) of *A. alternata* in different days of treatment.

	4-Hydroxybenzoic acid	Gallic acid	Syringic acid	Caffeic acid	Benzoic acid	Ferulic acid	Salicylic acid	Rosmarinic acid	Cinnamic acid	Total phenolic compound
	µg gFw^−1^									
Ctrl_Day 7	19.7 ± 3.2 a	0.19 ± 0.01 a	0.35 ± 0.01 a	0.5 ± 0.04 a	0.33 ± 0.01 c	V. L	V. L	0.12 ± 0.01 f.	1.36 ± 0.12 f.	22.61 ± 3.4 c
Pectin_Day 7	10.8 ± 1.9 b	V. L	V. L	V. L	0.15 ± 0.01 e	V. L	0.73 ± 0.03 b	0.8 ± 0.04 d	9.72 ± 1.7 d	22.24 ± 3.7 c
Ctrl_Day 14	5.68 ± 1.5 cd	V. L	V. L	V. L	V. L	V. L	V. L	0.96 ± 0.04 c	0.95 ± 0.004 g	7.66 ± 1.5 e
Pectin_Day 14	3.4 ± 1.1 de	0.16 ± 0.01 b	V. L	V. L	0.94 ± 0.01 b	V. L	1.16 ± 0.11 a	0.47 ± 0.02 e	6.89 ± 1.4 e	13.07 ± 2.6 d
Ctrl_Day 21	3.07 ± 1.15 de	V. L	V. L	0.15 ± 0.01 b	2.53 ± 0.12 a	0.33 ± 0.02 b	0.76 ± 0.06 b	1.19 ± 0.08 b	65.71 ± 6.5 a	73.76 ± 7.9 a
Pectin_Day 21	8.5 ± 1.5 bc	V. L	V. L	V. L	1.03 ± 0.09 b	0.1 ± 0.01 d	V. L	0.3 ± 0.012 e	48.77 ± 4.7 b	58.9 ± 6.3 b
Ctrl_Day 42	V. L	V. L	V. L	V. L	0.27 ± 0.02 d	0.59 ± 0.01 a	1.14 ± 0.1 a	3.65 ± 0.13 a	20.42 ± 2.7 c	6.07 ± 2.9 e
Pectin_ Day 42	1.81 ± 0.16 e	0.1 ± 0.01 c	V. L	V. L	2.05 ± 0.24 a	0.23 ± 0.02 c	0.63 ± 0.05 c	1.02 ± 0.07 c	71.27 ± 7.8 a	77.19 ± 8.3 a

Values are means of three replications ± standard deviation. Different letters show the significant difference with *p* ≤ 0.05 in one-way ANOVA and Duncan’s analyses. Means followed by the same letter in each column are not significantly different.

*V. L* very low, the content less than 0.1 µg gFW^−1^, *Ctrl* control (non-treated group).

### Data analysis using mathematical equations

**Figure 6:**
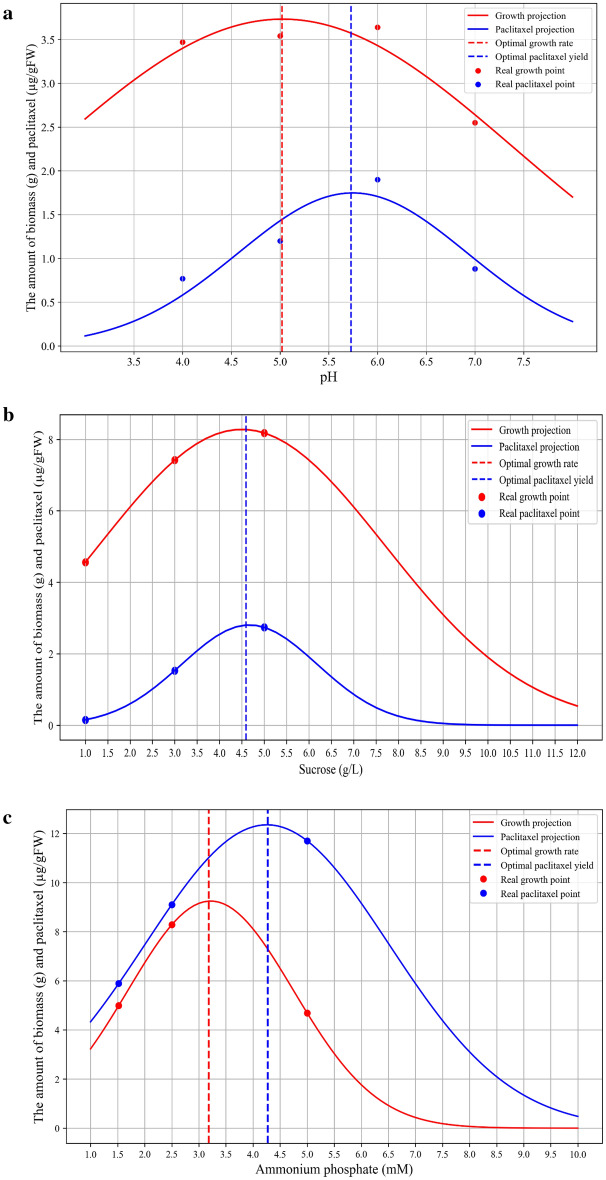
Optimum display of produced paclitaxel and growth in different concentrations of acidity (**a**), sucrose (**b**), and ammonium phosphate (**c**) by means of statistical analyses and Gaussian function.

As shown in Fig. 6, Gaussian function in Python software was used to calculate and draw plots. The intricate interplay between the points derived from dependent variables, namely the growth rate and paclitaxel yield, and independent variables (i.e., acidity, sucrose, and ammonium phosphate) was shown in the plots (Fig. 6a–c). The independent variables contribute a central role in determining the optimal conditions to maximize their effect. Analysis of the data using formulating and equations revealed that the optimum range of acidity for achieving maximum efficiency of *A. alternata* is around 5.0 to 5.7, where the highest levels of the dependent variables are observed. Furthermore, any deviation from these pH levels, whether towards alkaline or more acidic conditions, leads to a decrease in both the growth rate and paclitaxel yield (Fig. 6a).

Figure 6b clearly shows the different concentrations of sucrose as a decisive determinant for reaching the maximum of the dependent variable. The optimal concentration of sucrose for *A. alternata* is around 4.6% (w/v), resulting in a growth rate of 8.3 g flask^−1^ and a yield of 2.3 µg gFW^−1^, respectively. As expected, taxanes are not synthesized at concentrations of less than 1% and more than sucrose 9% (w/v) (Fig. 6b).

Figure 6c clearly shows that the concentration of ammonium phosphate 3.2 mM leads to a significant growth rate (9.3 g flask^−1^) (Fig. 6c). However, it is noticeable the highest paclitaxel yield is around 4.3 mM ammonium phosphate. Obviously, the low and high concentrations of ammonium phosphate have a negative influence on the growth rate or the efficiency of paclitaxel (Fig. 6c).

## Discussion

In recent years, researchers have alternatively employed endophytic fungi for a myriad of purposes, e.g. the production of paclitaxel. By persistent and repeated efforts, some genes of the taxane biosynthetic pathway are also known in fungi^25,32^. However, some isolates failed for the synthesis of paclitaxel or lost their ability to synthesize taxanes over time, leading to reject the potential of these fungi to produce taxanes^9^. In addition, investigations conducted in the past have demonstrated that among the extensive array of extracted endophytic strains, only some certain strains had the potential to synthesize taxane compounds^33^.

It should be noted that identifying strains requires considerable efforts with particular attention to keeping them in the fermentation cycle over long periods. Subsequent endeavors should focus on creating a simple optimal environment for the fermentation and production of secondary metabolites^34,35^, as formulating intricate combinations in the culture medium can be challenging in terms of both complexity and cost. Subsequently, the quantity of secondary metabolites should be also improved using various biotechnological methods. To prevent the strains from being limited or even losing the ability to synthesize molecules of interest in batch cultures, the establishment of alternating mycelial cultures, a prerequisite for maintaining long-term fermentation cycles, is essential. *A. alternata* was isolated from Iranian hazelnut plants in 2017 and initial studies indicated its potential for the taxane production. However, the lack of a stable mycelial line and an optimal environment made it difficult to verify whether these endophytic fungi would remain stable over time.

The acidity of the culture medium stands out as the most important factor affecting the productivity of the culture media and cell productivity. Acidity performs a pivotal role in the metabolism of microorganisms and influences various processes related to the synthesis of primary and secondary bioactive metabolites. Previous studies have shown that paclitaxel exhibits a high level of stability in a pH range of 3.0–5.0^36^. Notably, *Aspergillus terreus* has been demonstrated to produce substantial amounts of paclitaxel when cultured at pH 7.0 to 8.0^37^. Similarly, *Epicoccum nigrum* TXB502 and *Alternaria alternata* F3 have shown an optimal fermentation performance at pH 6.0^38,39^. In contrast, *Paraconiothyrium variabile* and *Epicoccum nigrum* exhibited the highest paclitaxel efficiency at a slightly lower pH of 5.0^40^. Nevertheless, numerous studies have demonstrated that endophytic fungi have the ability to ferment over a wide pH spectrum.

This research revealed that *Alternaria* fungus had the highest fermentation rate at pH 6.0, where the highest biomass content, cephalomannine, and paclitaxel yield were obtained. *Tian* et al., suggested that compounds such as paclitaxel, 10-deacetyl baccatin III, baccatin III, and N-benzoyl-3-phenylisoserine ethyl ester exhibit maximum stability under acidic conditions^41^. In addition, the molecular cloning of a gene related to the taxane biosynthetic pathway in *Taxus cuspidata* cells, e.g. O-phenylpropanoyl transferase enzymes, revealed that the optimum pH for the recombinant O-(3-amino-3-phenylpropanoyl) transferase falls in the range of neutral^42^. In particular, acidic conditions can activate silent gene clusters and thus increase the production and stability of taxoids. These results highlight the possible influence of acidic conditions in augmenting both taxoids and their overall yield.

It has been shown that fermentation and the production of secondary metabolites by endophytic fungi are strongly influenced by the nutrient sources of the fermentation medium^43^. The utilization of simple nutrients such as carbon sources proved to be not only economically viable but also environmentally friendly, contributing to the increase in the growth rate and secondary metabolites. Furthermore, the use of media with an appropriate carbon source improves fermentation, contributes to the stability of the endophytic fungi, and facilitates intermittent fermentation^44^.

According to the last stage of this study, the initial growth rate and taxoid yield of *A. alternata* in PDB medium were insufficient. Hence, six carbon sources, namely sucrose, glucose, sorbitol, malt extract, and mannitol, were used to improve the fermentation processes. *Sonaimuthu* et al. found sucrose (1% w/v) facilitated the maximum growth and paclitaxel production in *Pestalotiopsis oxyanthi* SVJM060^45^. To optimize the paclitaxel production in *Fusarium redolens*, sucrose 8% (w/v) was reported by *Garyali* et al., while the maximum paclitaxel yield of *Fusarium solani* was recorded in glucose 4% (w/v) in *Garyali* et al., work^46^. The most efficient source of carbon for the production of paclitaxel in *Alternaria tenuissima* was also glucose (8% w/v)^29,47^.

According to our results, only sucrose demonstrated significant effects on the biomass of *A. alternata*, which increased by more than 80% to fourfold compared to other carbon sources. Although sucrose does not lead to a significant boost in the quantity of taxane compared to other carbon sources, its effect on the biomass rate could potentially contribute to an overall improvement in the production efficiency of taxanes compared to other sources. The results indicated that fructose and sorbitol yielded the highest amounts of 10-deacetyl baccatin III and paclitaxel, respectively. Fructose and sorbitol are examples of polyalcohols and sugar alcohols which serve as carbon and energy sources for specific microorganisms. Research has demonstrated that certain microorganisms possess the ability to metabolize fructose and break it down into dihydroxyacetone phosphate and glyceraldehyde-3-phosphate^41^. These metabolites can then serve as precursors for the synthesis of secondary metabolites.

The interplay of some media factors is a decisive factor in increasing the production of secondary metabolites by fungi. The use of suitable nitrogen sources in combination with acidity and carbon sources can be a decisive factor in enhancing the yield of microorganisms. As documented in studies by *Xu* et al., and *Garyali* et al., ammonium nitrate has been shown to be the most effective nitrogen source for *Fusarium redolens* and *Fusarium maire* K178^46,47^.

According to *El-Sayed* et al., sodium nitrate was identified as the most effective nitrogen source for *Aspergillus fumigatus*. However, ammonium nitrate led to an increased taxane yield in the fungus of up to 327 mg L^−1^. Conversely, ammonium dihydrogen phosphate was found to enhance the dry weight rate in *Alternaria tenuissima*, while ammonium nitrate specifically influenced the quantity of taxanes. *El-Sayed* et al., reported that different fungal species respond differently to nitrogen sources and the selection of a nitrogen substrate plays an important role in determining the amount of paclitaxel^29^. Our results showed that the maximum growth rate and paclitaxel yield by *A. alternata* were produced when (NH_4_)_2_HPO_4_ (2.5 mM) was used. There is compelling evidence indicating that there are 20 enzymatic steps involved in the biosynthetic pathway of taxoids, utilizing the common terpenoid precursor, geranylgeranyl diphosphate^48–50^.

The levels of amino acids and alkaloids are intricately regulated by the interactive effects of nitrogen supply and carbon status. There is substantial evidence suggesting that nitrogen, particularly in the form of ammonium, exerts significant impacts on organisms compared to nitrate. However, distinct studies have demonstrated that urea enhances the synthesis of most amino acids^51^. *Wang* et al., noted that NH_4_ as the nitrogen source positively influenced the accumulation of amino acids, with particular emphasis on thiamine, glutamate, and arginine, contrasting with the effects observed with NO_3_^52^. Furthermore, our results highlight that not only the nitrogen source, but also accompanying elements such as phosphate, potassium, and sulfate, play significant roles in influencing the amino acid and alkaloid profiles of *Alternaria*.

The utilization of biotic elicitors, e.g. pectin and alginate, to stimulate the formation of secondary metabolites has proven to be an important promotion strategy. This approach has not only proven effective in reducing the processing time required to achieve high product concentrations and productivity, but it is also eco-friendly. In addition, signaling molecules such as poly- or oligosaccharides are regulators of biosynthetic pathways^53,54^.

Recent reports indicate that the growth of organisms is inhibited in treated cultures with polysaccharides under in vitro conditions; however, pectin and salicylic acid enhance the biosynthesis of secondary metabolites^55^. Simic et al., reported that pectin not only promoted the accumulation of secondary metabolites in *Hypericum perforatum* cells but also led to a significant downturn in total phenolic and flavonoid production from day 14 to day 21^56^. The accumulation of oleanolic acid in cell suspension cultures of *Calendula officinalis* L. was found to be facilitated by pectin^57^. Further investigations on fungi have shown that alginate increased mycophenolic acid production in *Penicillium roqueforti*^58^, while gellan gum promoted the cyclosporine yield^59^.

In cell suspension cultures of *Vitis vinifera*, *Cai* et al., demonstrated that the addition of pectin promotes both cell growth and the production of anthocyanins^60^. In addition, in cell cultures of other plant species such as *Cocos nucifera*, chitosan treatment was observed to promote the production of various secondary metabolites, including phenolic acids^61^. According to prior reports, polysaccharides are a markedly effective inducer of naphthodianthrone production in cells of *Hypericum perforatum*^62^. Based on the latest research, *Aspergillus fumigatus* TXD105–GM6 and *Alternaria tenuissima* TER995–GM3 which were immobilized in calcium alginate beads produced paclitaxel up to 902 µg L^−1^ and 529 µg L^−1^ levels, respectively^63^.

Based on the results of the present study, the temporal dynamics observed in the mycelial growth and taxane production highlight intriguing patterns that shed light on the intricate relationship between the triggers and the fungus. The increase of mycelia growth rate was induced by pectin up to day 21, especially in 10-deacetyl baccatin III (12-fold more than the control on day 21), suggesting that pectin acts as a beneficial elicitor. Since pectin is a component of plant cell walls, these findings may elucidate the augmentation in the mycelial biomass.

Phenolic acids are molecules derived from organisms that have a high antioxidant capacity, mainly due to their hydroxyl groups. *Domínguez Avila* et al., indicated that phenolic acids, especially gallic acid, were significantly influenced by the presence of pectin^64^. The accumulation of phenolic acids especially 3-O-glucosyl resveratrol in *Vitis vinifera* cultures^60^, both within the cultures themselves and in the culture medium, was notably enhanced by chitosan, alginate, and gum arabic. According to researchers, it has been demonstrated that pectin interacts with phenolic compounds in a way that affects antioxidant activity^65^. The results of the present study unveil intriguing insights into the taxane and phenolic acid profiles following 21 days, indicating a notable deviation towards the biosynthetic pathways of taxanes. Additionally, pectin may have contributed to the production of taxanes by nourishing the mycelia, strengthening their production, and influencing antioxidant activity. Beyond on 21th day of fermentation, a shift in the metabolic pathways of *A. alternata* suggests a change in its regulatory response. The substrate, pectin, might no longer support optimal taxane production, leading to a redirection of biosynthetic pathways toward the production of alternative compounds e.g. terpenoids.

The statistical models derived from our analysis emphasize the importance of optimal acidity in enhancing efficiency. However, it is noteworthy that cellular responses to different acidity levels may diverge among different cells. Furthermore, the normal distribution plot of carbon and nitrogen sources aptly illustrates that a shortage of available nutrient sources can affect both growth rate and paclitaxel yield. Conversely, the excess of nutrient sources can disrupt the intracellular balance, leading to cellular stress and eventual death.

## Conclusion

Based on the results of the research conducted on *A. alternata* fungi, maintaining the stability of the fungi is considered essential. Through a prolonged period, we were able to demonstrate the stability of *A. alternata* and the production of taxanes by establishing a mycelium line. Subsequently, parameters such as acidity, carbon, and nitrogen sources were optimized to achieve the optimal rate of growth and taxane production. It is noteworthy that the growth of fungi and taxanes, e.g. paclitaxel, was initially minimal in the PDB environment. However, significant improvement was observed in both the growth rate and taxane production, e.g. 10-deacetyl baccatin III, baccatin III, 7-epi-10-deacetyl paclitaxel, paclitaxel, and 7-epi paclitaxel, after the optimization. Finally, the environmentally friendly stimulus was applied to enhance the quantity of the parameters of interest. Findings from the research are a prerequisite for future studies and can also be utilized in industry as a guidance line for large-scale production of taxoids, which in turn can be used in the treatment of a wide range of cancers.

## Materials and methods

### Chemicals

All chemical substances were obtained from Merck (Germany). Taxane standards were sourced from Sigma-Aldrich and ChromaDex (USA).

### Initiation of suspension cultures

*Alternaria alternata* KT223359.1 has previously been isolated from *C. avellana*^66^. The first report on this fungus was originally described by *Esenbeck* et al.,^67^ and *Fries* et al.^68^, although it has also recently been isolated from other plants e.g. beans, collected from Mozambique^69^. The fungus was grown on potato dextrose agar (PDA) medium (Q-Lab Corporation), pH 5.5, at darkness, 25 ± 2 °C. The cultures were periodically refreshed every 10 days. Suspension cultures were established by immersing fresh mycelia in potato dextrose broth (PDB) (Q-Lab Corporation), pH 5.5, on a reciprocal shaker, 110 rpm. Sub-cultures were conducted every seven days and after 20 sub-cultures, a line with a constant growth was obtained.

### Optimizing pH, carbon sources, and nitrogen sources

A multi-step investigation was accomplished to identify the most suitable carbon and nitrogen forms and concentrations. In the first step, the effect of pH 4.0, 5.0, 6.0, and 7.0 on mycelial growth in PDB was examined for one week. According to the results, pH 6.0 had the most significant positive effect on the growth rate and the yield of taxanes and was used as the optimum pH in further experiments.

To select the best carbon source, the fungus was cultured in simple media containing aqueous solutions (3% w/v) of malt extract, sucrose, sorbitol, glucose, fructose, and mannitol (Merck Corporation). Based on the results of this experiment, sucrose was selected as the best carbon source and in the next step was applied at different concentrations i.e., 1, 3, and 5% (w/v), where the latter showed the highest increasing impact on the mycelia growth and taxane yield.

The effects of different nitrogen sources were evaluated by culturing the fungus in media all containing 5% sucrose, pH 6.0 and (NH_4_)_2_HPO_4_, CH_4_N_2_O, NH_4_NO_3_, (NH_4_)_2_SO_4_, and KNO_3_ (Merck Corporation), 2.5 mM of each. Based on the result of this step ammonium phosphate was selected as the best nitrogen source. Furthermore, it was applied in different concentrations i.e., 1.25, 2.5, and 5 mM, where ammonium phosphate with the 2.5 mM concentrations showed the highest improving effect on the growth and taxane production.

In all steps, the mycelia were harvested, washed thoroughly under reduced pressure, accurately weighed, rapidly frozen with liquid nitrogen, and kept at − 80 °C before HPLC analysis and taxane determination.

### Qualitative and quantitative analysis of taxanes

Taxanes were sequentially extracted with MeOH and CH_2_Cl_2_ and were analyzed by HPLC as previously explained^70^. Each peak was first determined based on its RT and further confirmed by injecting its corresponding standard for validation.

An Agilent 6410 HPLC systems coupled to triple quadrupole ion trap mass spectrometers equipped with electrospray ions were used to initially analyze the samples. An Eclipse C18 column was used (3.5 lm particle size, 100 mm length, and 4.6 mm width). Solvent flow rates and injection volumes were 350 µL min^−1^ and 50 µL min^−1^, respectively. In mobile phase A, distilled water: MeOH was used at 70:30 (v/v), while mobile phase B was composed of 0.1% formic acid-containing MeOH. For five minutes, an isocratic flow (1 mL min^−1^) was employed. A linear gradient of 100% A was then applied to 100% B for 25 min, followed by an isocratic gradient of 100% B for 10 min (a total run time of 40 min). In production mode, mass spectra were acquired. ESI/MS was used to verify the presence of taxanes in samples using structurally diagnostic ions in LC–MS spectra^71^.

### Determination of total alkaloid content

The samples were homogenized in EtOH 96% and agitated overnight. Following centrifugation at 10,000×*g* for 10 min, the supernatant was heated at 80 °C for one hour and desiccated at room temperature. The remaining residue was subsequently treated with H_2_SO_4_ 1.0 M and extracted with Et_2_O. The pH of the aqueous phase was adjusted to 9–12 by adding NH_4_OH 25% and then was extracted by Et_2_O. The absorbance was measured at 254 nm utilizing a spectrophotometer (GBC, Cintra 6, Australia). Colchicine was used as a standard^72^.

### Extraction of pectin from *Corylusavellana* cells

The cells were homogenized with absolute EtOH and filtered. The caked cells were then treated with a mixture of MeOH: CHCl_3_ (2:1), overnight, followed by filtration. Acetone was added to the residue and filtered after 1 h. After drying the residue (wall powder), pectin was subsequently extracted with hot ammonium oxalate (20 mM) and NaOH (0.1 M) and then dialyzed (12–14 cut off) against distilled water overnight. During dialysis, the water was changed every 2 h. *A. alternata* was exposed to the extracted pectin with a concentration of 0.125 µM. Mycelia were collected on days 7, 14, 21, and 42.

### Molecular weight determination of pectin

A solution comprising 2 mg mL^−1^ of pectin in distilled water was prepared and exposed to microwave heating for 30 s. After preparation, a 3.0 μm cellulose membrane (Whatman International) was used to filtrate the pectin solutions. A multi-angle static laser light scattering system (HELEOS; Wyatt Technology Corp, Santa Barbara, CA, USA) connected to a refractive index detection system (HPSEC-MALLS-RI) and a TSK G5000PW column (7.5 × 600 mm; Toso Biosep, Montgomeryville, PA, USA) was employed to determine molecular weight properties. The mobile phase with a flow rate of 0.4 mm min^−1^ was employed, comprised of 0.15 M NaNO_3_ and 0.02% NaN_3_. For the calculation of average molecular weight (Mw) (Wyatt Technology Corp.), ASTRA software version 3.5 (http://www.wyatt.com/products/software/astra.html, Wyatt Technology Corp., Santa Barbara, CA) was utilized.

### Evaluation of total protein and soluble amino acid levels

The assessment of total protein and soluble amino acids content was carried out the Bradford method, employing bovine serum albumin as a standard^73^. Soluble amino acids were extracted in EtOH 80% and treated with a mixture containing ortho-phthalaldehyde (OPA), MeOH, borate buffer, and β-Mercaptoethanol, and then analyzed by HPLC system (Agilent Technologies 1260 infinity, USA). Detailed elution procedures can be found elsewhere^74^.

### The quantitation of terpenoid levels

Samples were homogenized in MeOH 95% and centrifuged at 4000×*g* for 15 min. CHCl_3_ was added to the suspensions and vortexed, and then H_2_SO_4_ was added. The solution was incubated at room temperature for two hours. Following this, the supernatant was decanted. Subsequently, MeOH 95% was added to the precipitate, and absorption was assessed using a spectrophotometer at 538 nm (GBC, Cintra 6, Australia). Menthol (C_10_H_20_O) was used as the standard^75^.

### Qualitative and quantitative analysis of phenolic compounds

For the extraction and determination of phenolic acids, the mycelia were extracted with a mixture of MeOH and HOAc (99:1, v/ v) and centrifuged at 12,000×*g* for 15 min. The supernatant was dried, re-solved in methanol, filtered, and injected into an HPLC system (Waters, e2695, USA). The system was equipped with a C18 column (Perfectsil Target ODS3, 5 μm, 250 × 4.6 mm, MZ-Analysentechnik, Mainz, Germany) and UV detector. Flow rates of 1 mL min^−1^ were used to elute phenolic acids with HOAc 97% as solvent A and MeOH as solvent B, under the following gradient conditions: 0–2 min, 5% B; 2–10 min, 25% B; 10–20 min, 40% B; 20–30 min, 50% B; 30- 40 min, 100% B; 40–50 min, 5% B, 278 and 300 nm^76^.

### Statistical analysis

All experimental procedures were carried out with a minimum of three independent replicates. The statistical analysis of the data was conducted through one-way ANOVA. The significance of the differences was assessed with Duncan test, *p* ≤ 0.05. GraphPad Prism version 9. 5. 1 for Windows (Graph Pad software, San Diego California USA, URL: www.graphpad.com) was applied to design plats.

Python software version 3. 10. 7 (Python Software Foundation, Beaverton, OR, URL: https://www.python.org/) was employed for a more detailed analysis of data. The scipy.optimize library and the Gaussian function were utilized to determine optimal parameters and data fitting. Additionally, the matplotlib.pyplot library was employed for creating plots.

## Data Availability

The datasets generated during and/or analyzed during the current study are available from the corresponding author on reasonable request.
